# Effects of tibial torsion on distal alignment of extramedullary instrumentation in total knee arthroplasty

**DOI:** 10.3109/17453674.2013.792032

**Published:** 2013-05-31

**Authors:** Gianluca Cinotti, Pasquale Sessa, Antonello Della Rocca, Francesca Romana Ripani, Giuseppe Giannicola

**Affiliations:** Department of Anatomy, Histology, Medico Legal and Orthopaedic Science, University La Sapienza, Rome, Italy.

## Abstract

**Background and purpose:**

Whether tibial torsion affects the positioning of extramedullary instrumentation and is a possible factor in malalignment of the tibial component in total knee arthroplasty (TKA) is unknown. We assessed the influence of tibial torsion on distal alignment of extramedullary systems for TKA, using the center of the intermalleolar distance as anatomical reference at the ankle joint.

**Patients and methods:**

We analyzed CT scans of knee and ankle joints of 50 patients with knee osteoarthritis (mean age 73 years, 52 legs). The tibial mechanical axis was identified and translated anteriorly at the level of the medial one-third (proximal AP axis 1), at the medial border of the tibial tuberosity (proximal AP axis 2), and at the level of the talar dome (distal AP axis). The center of the intermalleolar distance and the width of the medial and lateral malleolus were calculated. The proximal AP axes 1 and 2 were translated at the level of the ankle joint and any difference between their alignment and the distal AP axis was calculated as angular and linear values.

**Results:**

The center of the ankle joint was located, on average 2 mm medial to that of the intermalleolar distance. The distal AP axis was externally rotated by 18° and 27° compared to the proximal AP axes 1 and 2, respectively. Overall, the center of the ankle joint was shifted laterally by 9–11 mm with respect to the proximal AP tibial axes.

**Interpretation:**

To avoid a varus tibial cut in TKA, extramedullary alignment systems should be aligned more medially at the ankle joint than previously thought, due to the effect of tibial torsion and—to a lesser extent—to the different malleolar width.

Experimental and clinical investigations have shown that proper implant positioning may reduce the incidence of aseptic loosening and increase the longevity of TKA (Hsu et al. 1989, Green et al. 2002, Perillo-Mercone and Taylor 2007, Fang et al. 2009). However, tibial alignment with current extremedullary or intramedullary instrumentations is not entirely satisfactory since varus-valgus malalignment greater than 3° has been reported in 2–40% of cases (Teter et al. 1995, Reed et al. 2002, Mihalko and Krackow 2006, Chiu et al. 2008).

Extramedullary systems need to be aligned in the coronal and sagittal planes using anatomical landmarks in the proximal and distal tibia. While several studies have assessed the most appropriate anatomical landmarks in the proximal tibia (Akagi et al. 2004, Huddleston et al. 2005, Aglietti et al. 2008, Cobb et al. 2008, Lützner et al. 2010), anatomical references at the distal tibia and ankle joint have not been thoroughly investigated. In particular, with the exception of one study addressing the accuracy of palpable tendons as anatomical landmarks for the center of the ankle joint (Schneider et al. 2007), no other investigations have substantiated the reliability of the reference points currently used in TKA (Akagi et al. 2005, Mizu-uchi et al. 2006, Lützner et al. 2010).

A major issue in the correct alignment of an extramedullary guide at the ankle joint is external tibial torsion, i.e. axial rotation of the tibia along its longitudinal axis, which causes an external rotation of the distal tibial epiphysis relative to the proximal one (Takai et al. 1985, Eckhoff and Johnson 1994, Akagi et al. 2005, Mizu-uchi et al. 2006). As a result, tibial torsion leads to a lateral shift of the anterior projection of the center of the ankle joint, and if this translation is not taken into account during the alignment of the extramedullary guide, a varus tibial cut is likely to occur (Akagi et al. 2005). However, the extent to which tibial torsion may affect the position of the extramedullary guide at the level of the ankle joint is unknown.

We investigated whether the center of the intermalleolar distance, or a definite distance from it, overlaps the center of the ankle joint and can be used as an anatomical landmark for distal alignment of extramedullary systems in TKA. Our hypothesis was that when the center of the intermalleolar axis is used as a reference point to align the distal extramedullary guide, the effects of tibial torsion on the coronal alignment of the ankle joint must be taken into account to avoid a malalignment of the tibial component.

## Patients and methods

We analyzed CT scans of the knee and ankle joints of 56 patients with knee osteoarthritis scheduled for TKA. 6 patients who had postraumatic deformity of the femur, tibia, or ankle joint were excluded. CT studies of the remaining 50 patients (28 males, 52 legs) with a mean age of 73 (42–79) years were done. The investigation was performed according to institutionally approved guidelines after having received informed consent from all the patients.

### CT study

We used a leg holder to keep the lower limb in neutral rotation. A 64-row multidetector CT scanner (Lightspeed VCT; GE Medical Systems, Milwaukee, WI) was used. The diagnostic protocol included a section thickness of 0.625 mm. CT data sets were transferred to a dedicated workstation for processing (Advantage Windows 4.4; GE Healthcare Technologies, Buckinghamshire, UK). Reconstructed 3-D images available for the analysis included multiplanar reformation (MPR) images. 3-D CT images were first used to identify the tibial mechanical axis. Axial scans were then used to generate the anterior projection of the mechanical axis in the proximal and distal tibia.

### Anatomical references in the proximal tibia (Figure 1)

The projection of the tibial mechanical axis on the anterior cortex was calculated on an axial scan, perpendicular to the sagittal tibial axis, 10 mm caudally to the lateral tibial plateau, that is, at the level at which the proximal tibial cut is usually performed in primary TKA. On this axial scan, we first transposed the projection of the tibial tuberosity (TT) including the medial and lateral border, the middle point, and the medial one-third. On the same axial scan, we transposed the femoral transepicondylar axis and connected its middle point to the medial one-third of the TT (proximal AP axis 1) (Lützner et al. 2010). As the medial border of the TT is also used as proximal reference for tibial rotation alignment (Akagi et al. 2004), a second AP axis was considered—that is, a line connecting the PCL insertion at the posterior tibial notch with the medial border of the TT (proximal AP axis 2) (Akagi et al. 2004).

**Figure 1.**
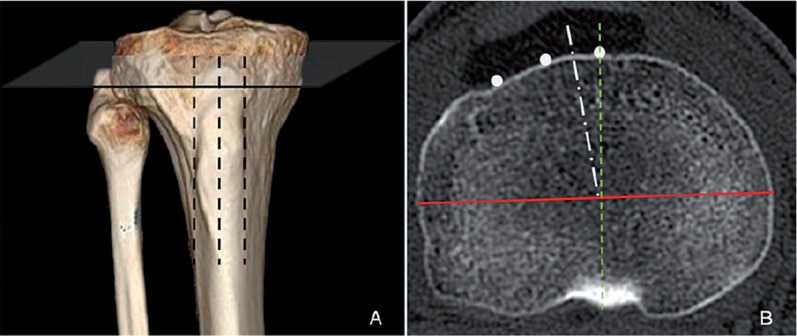
A. 3-D CT scan showing detection of the medial and lateral border and the middle of the tibial tuberosity (TT) and the level at which the proximal AP tibial axes were identified. B. Axial CT scan illustrating the projection of the TT (filled circles) and of the femoral transepiconylar axis (FTA) (red line). The white dash-dot line represents AP axis 1, connecting the middle of the FTA with the medial one-third of the TT. The green dotted line represents AP axis 2, connecting the posterior tibial notch with the medial border of the TT (Akagi line).

### The center of the intermalleolar axis and of the ankle joint (Figure 2)

An axial scan was selected at the level of the ankle joint and the intermalleolar axis was identified as the distance connecting the external cortex of the medial and lateral malleoli. The width of medial and lateral malleolus was also recorded. On the same scan, we calculated the center of the talar dome and its anterior projection (distal AP axis). The latter was determined by identifying the middle of the talar dome in its anterior and posterior portions and connecting the 2 points. We determined the relationship between the center of the intermalleolar axis and that of the ankle joint.

**Figure 2.**
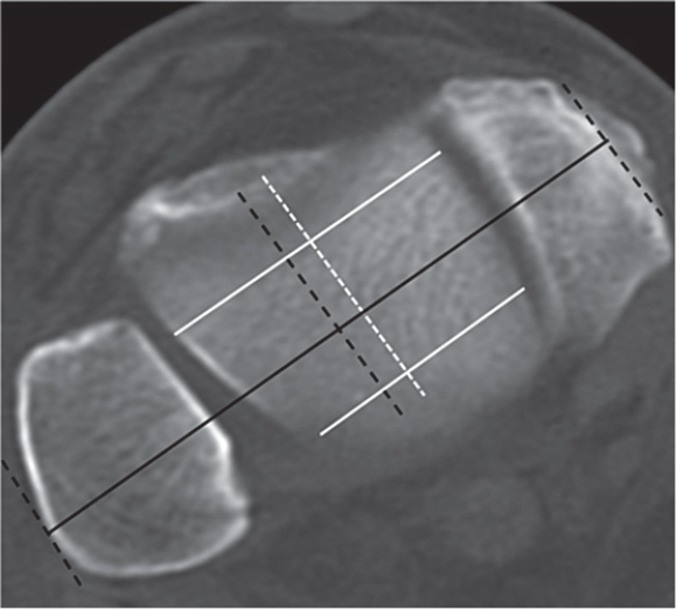
Axial CT scan depicting the intermalleolar distance (continuous black line) and its anterior projection (dotted black line). The white dotted line represents the center of the talar dome originating from the connection of 2 pints located in the middle of its anterior and posterior region (white continuous lines). Note that the center of the intermalleolar distance (dotted black line) is translated laterally with respect to the center of the talar dome.

### Correlation between the proximal and distal anatomical references (Figure 3)

The proximal AP axes 1 and 2 were transposed at the level of the ankle joint in order to visualize the proximal and the distal AP axes in the same axial scan. We then calculated the angle between the proximal and distal AP axes and the distance between them. A trigonometric analysis was done to assess the effects of the different rotational alignments of proximal and distal AP axes on the coronal alignment of the tibial cut [cos γ = (b^2^ + a^2^ – c^2^) / 2ab]. The reliability of measurements was assessed twice, the first time during the study and the second time 4 weeks after its completion, in 35 randomly selected patients. Intra-observer and inter-observer reliability were assessed using intraclass correlation coefficients (ICCs).

**Figure 3.**
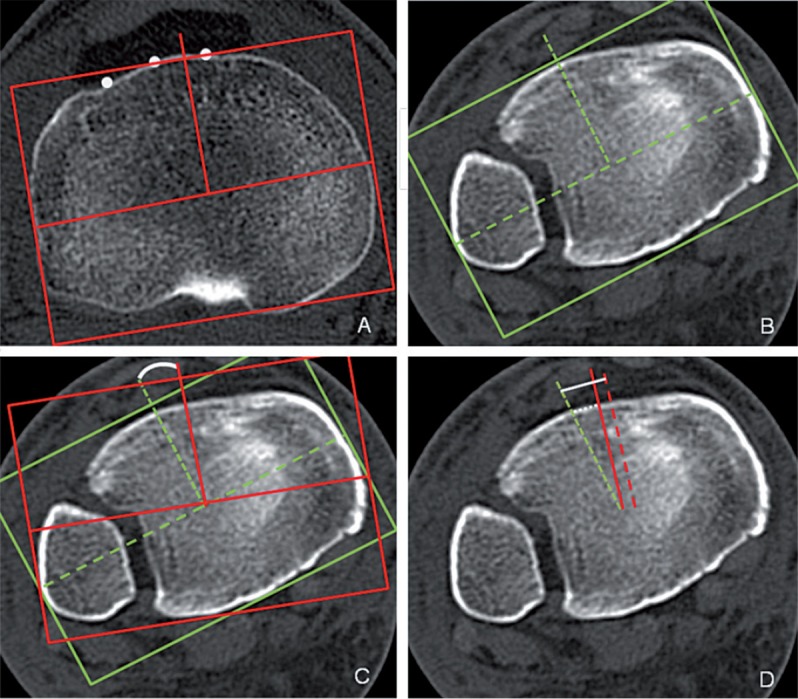
A. Axial scan showing proximal AP axis 1 passing through one-third of the tibial tuberosity (TT). B. Distal AP axis, i.e. the projection of the center of the ankle joint. C. Axial scan showing the mismatch in rotation alignment between proximal AP axis 1 (continuous red line) and the distal AP axis (dotted green line), the latter being externally rotated with respect to the former. D. Axial CT scan showing the extent to which, on average, the reference point of the ankle joint should be translated medially to compensate for tibial torsion (white dotted line) and for tibial torsion plus the difference in malleolar width (white continuous line).

### Statistics

The Lilliefors (Kolmogorov-Smirnov) normality test was performed for all the variables assessed. All variables showed a normal distribution (p = 0.2). Mean, SD, range, and 95% confidence interval (CI) were calculated for all the variables assessed. A linear correlation analysis and a linear regression analysis were performed between the variables analyzed and tibial length. SPSS version 17.0 for Windows was used.

## Results

The center of the intermalleolar distance did not overlap the center of the talar dome, but it was shifted laterally, on average, by 2.2 mm (range: 1.4–3.2 mm; SD 1.4; CI: 1.4–3.1 mm). This discrepancy was related to the different widths of the lateral and medial malleolus, which were, on average, 17 mm (range: 15–19 mm; CI: 16–17 mm) and 12 mm (range: 9–16; CI: 12–13 mm), respectively (p < 0.001).

The AP axis of the ankle joint was externally rotated compared to the proximal AP axes 1 and 2, on average by 19° (SD 9.5) and 27° (SD 12.4), respectively (Figure 3 and Table). The average distance between the AP axis of the ankle joint and proximal AP axes 1 and 2 was 6.5 mm (SD 3.6) and 8.7 mm (SD 3.6), respectively (Table 1). Intra-observer and inter-observer reliability was 0.92 and 0.96, respectively.

**Table 1.** Relationship between the center of the ankle joint, the intermalleolar distance, and proximal AP axes

	Mean	Range	SD	95% CI
Center of ankle joint versus center of the intermalleolar distance, mm **^a^**	2.0	0–5.7	1.4	1.4–2.6
AP axis of ankle joint versus				
AP axis 1, degrees **^b^**	19	3.3–38	9.5	16–22
AP axis 1, mm **^b^**	6.5	0.6–15	3.6	5.5–7.7
AP axis 2, degrees **^c^**	27	5.0–40	12	25–30
AP axis 2, mm **^c^**	8.7	1.1–20	6	6.8–9.3
a + b **^d^**	8.7	2.5–19	3.3	7.4–11
a + c **^e^**	11	3.1–20	2.9	10–12

**^a^** difference between the center of the intermalleolar distance and the center of the ankle joint. **^b^** external rotation of the AP axis of the ankle joint with respect to AP axis 1, expressed in degrees and mm. **^c^** external rotation of the AP axis of the ankle joint with respect to AP axis 2, expressed in degrees and mm. **^d^** lateral translation (in mm) of the AP axis of the ankle joint with respect to proximal AP axis 1 (in mm) plus (a). **^e^** lateral translation (in mm) of the AP axis of the ankle joint with respect to proximal AP axis 2 (in mm) plus (a).

When the difference between the center of the intermalleolar distance and that of the ankle joint was added to the difference between the proximal and distal AP axes, the center of the ankle joint shifted laterally—compared to proximal AP axes 1 and 2—on average by 8.7 mm (SD 3.3) and 11 mm (SD 2.9), respectively (Table). Trigonometric analysis showed that a lateral displacement of 2 mm caused a varus cut of 0.88°, which meant that a lateral translation of 10 mm led to a varus alignment of tibial cut of 4.4°.

## Discussion

Extramedullary systems are frequently used in TKA. Compared to intramedullary systems, they provide similar results in terms of coronal alignment where there is normal tibial morphology (Dennis et al. 1993, Ishii et al. 1995, Maestro et al. 1998) and they are more accurate when tibial bowing or postraumatic deformities are present (Dennis et al. 1993, Bono et al. 1995, Nagamine et al. 2000, Ko et al. 2001, Chiu et al. 2008). Furthermore, they avoid violation of the medullary canal, with the potential benefit of reducing the risk of pulmonary embolism (Morawa et al. 1996, Church et al. 2007). The major limitation of extramedullary systems is that their alignment relies on the identification of proximal and distal anatomical landmarks, the reliability of which is not well established (Akagi et al. 2004, Huddlestone et al. 2005). In particular, while several investigations have assessed the reliability of tibial tuberosity, posterior cruciate ligament insertion, intercondylar eminence, and center of tibial plateaus for proximal alignment of extramedullary guide and axial rotation of tibial components (Akagi et al. 2004, Parker Vail and Lang 2006, Aglietti et al. 2008, Cobb et al. 2008, Lützner et al. 2010), there have been very few studies on reference points in the distal tibia and ankle joint. It has been recommended that the center of the ankle joint should be in line with the first or second metatarsal (Teter et al. 1995, Akagi et al. 2004, Huddlestone et al. 2005), with a point located 3–5 mm medial to the intermalleolar distance (Reed et al. 2002, Huddlestone et al. 2005, Parker Vail and Lang 2006), with the anterior tibial tendon 5 cm above, or at the level of, the joint line (Schneider et al. 2007). However, to our knowledge, none of these recommendations came from anatomical studies in which the accuracy of these reference points was assessed. In the only investigation that concentrated on this issue, Schneider et al. (2007) found that the extensor hallucis longus (EHL), by lying close to the center of the ankle joint, was the most accurate anatomical landmark, while the tibialis anterior tendon—even though more easily palpable—was located about 1 cm medial to the center of the ankle. However, as the authors pointed out, it can be difficult to identify the EHL in obese patients. Furthermore, while pronation had little effect on the position of the EHL, supination caused tendon displacement of greater than 1 cm.

We found that due to the axial torsion of the tibia along its longitudinal axis, the anterior projection of the center of the ankle was externally rotated compared to the AP axis of the proximal tibia—by an average of 19–27°, depending on whether the medial one-third, the medial border, or the TT was used as the proximal anatomical reference. Tibial torsion is a well known anatomical feature of the tibia; it was first described in the early 1900s and was further defined as soon as CT investigations became available (Le Damany 1909, Jakob et al. 1980, Takai et al. 1985). However, the effects of tibial torsion on the alignment of extramedullary systems in TKA have not been investigated in any detail. In the only study that, to our knowledge, has addressed this point, Mizu-Uchi et al. (2006) found that the AP axis of the ankle joint was externally rotated by 3.6–20° depending on which AP axis of reference was used at the proximal tibia. However, the extent to which the extramedullary guide should be translated medially—with respect to the middle of the intermalleolar distance, in order to compensate for external rotation of the ankle and to avoid a tibial cut in varus—was not assessed. In the present study, using the same AP axis in the proximal tibia, we found a greater mismatch between the proximal and the distal AP axes than that reported by these other authors, possibly due to racial differences in tibial torsion between Asian and Caucasian subjects. Furthermore, we found that to avoid coronal malalignment, the extramedullary alignment guide should be translated medially by 9–11 mm. This translation, which is greater than previously reported (Dennis et al. 1993, Reed et al. 2002, Parker Vail and Lang 2006), is necessary to compensate either for the different rotational alignment of the proximal and distal tibia, i.e. for tibial torsion, and for the reduced thickness of the medial malleolus compared to the lateral one, which was found to cause a further lateral translation of the center of the intermalleolar axis with respect to the center of the ankle joint. We found that a translation of the distal extramedullary guide of 2 mm caused a coronal tilt of tibial cut of 0.88°. As a result, by using the standard reference at the ankle joint, i.e. 3–5 mm medial to the intermalleolar axis (Dennis et al. 1993, Reed et al. 2002, Parker Vail and Lang 2006), and a proximal AP axis in-between the medial border and one-third of the TT, a tibial cut in varus of 2–3° might occur. The varus malalignment might be aggravated further when a lateral alignment of the distal extramedullary guide is associated with a tibial cut performed with a posterior slope, since in this case a postero-medial tibial slope rather than a posterior tibial slope is generated (Mizu-uchi et al. 2006).

Several investigations analyzing implant alignment in TKA performed with extramedullary systems have found that varus cut is the most frequent error in alignment of the tibial component (Dennis et al. 1993, Teter et al. 1995, Reed et al. 2002). Our results have clearly shown that this error is likely to occur if the extramedullary guide is not translated medially, with respect to the middle of the intermalleolar distance, and that this medial translation is greater when the medial border of the TT is used as proximal reference rather than the medial one-third of the TT. However, we also found that tibial torsion showed a wide range of variation in the series analyzed, a result that may further explain the higher rate of outliers found in standard TKA than in navigated TKA (Chiu et al. 2008).

One limitation of our study was that we used only 2 of the proximal AP axes commonly used in TKA, and the results could be different when other AP axes are considered in the proximal tibia. Similarly, at the level of the ankle joint, the center of intermalleolar axis was used as distal reference for the extramedullary guide since this is one of the most commonly used anatomical landmarks with extramedullary systems. However, our findings do not necessary apply to surgical procedures in which palpable tendons or other bony landmarks are used. A further limitation was that in 2 out of 50 cases we measured the right and left limb in a subject, with a potential risk of data distortion due to intraclass correlation. However, we believe that this risk should be reasonably low since the proportion of independent measurements was 92%.

In conclusion, tibial torsion and (to a lesser extent) the reduced thickness of the medial malleolus compared to the lateral one, causes a mismatch in the coronal alignment between AP axes of the proximal and distal tibial epiphysis, where the center of the intermalleolar distance is shifted laterally by about 9–11 mm relative to the proximal AP axes. As a result, to avoid tibial cut in varus, the extramedullary alignment system should be translated medially by about 9–11 mm, a distance that is greater than previously recommended. Further investigations should determine whether the rate of coronal malalignment of the tibial component may be reduced by using these reference points for distal alignment of extramedullary systems.

## References

[CIT0001] Aglietti P, Sensi L, Cuomo P, Ciardullo A (2008). Rotational position of femoral and tibial components in TKA using the femoral transepicondylar axis. Clin Orthop.

[CIT0002] Akagi M, Oh M, Nonaka T, Tsujimoto H, Asano T, Hamanishi C (2004). An anteroposterior axis of the tibia for total knee arthroplasty. Clin Orthop.

[CIT0003] Akagi M, Mori S, Nishimura S, Nishimura A, Asano T, Hamanishi C (2005). Variability of extraarticular tibial rotation references for total knee arthroplasty. Clin Orthop.

[CIT0004] Bono JV, Roger DJ, Laskin RS, Peterson MG, Paulsen GA (1995). Tibial intramedullary alignment in total knee arthroplasty. Am J Knee Surg.

[CIT0005] Chiu KY, Yau WP, Ng TP, Tang WM (2008). The accuracy of extramedullary guides for tibial component placement in total knee arthroplasty. Int Orthop.

[CIT0006] Church JS, Scadden JE, Gupta RR, Cokis C, Williams KA, Janes GC (2007). Embolic phenomena during computer-assisted and conventional total knee replacement. J Bone Joint Surg (Br).

[CIT0007] Cobb JP, Dixon H, Dandachli W, Iranpour F (2008). The anatomical tibial axis: reliable rotational orientation in knee replacement. J Bone Joint Surg (Br).

[CIT0008] Dennis DA, Channer M, Susman MH, Stringer EA (1993). Intramedullary versus extramedullary tibial alignment systems in total knee arthroplasty. J Arthroplasty.

[CIT0009] Eckhoff DG, Johnson KK (1994). Three-dimensional computed tomography reconstruction of tibial torsion. Clin Orthop.

[CIT0010] Fang DM, Ritter MA, Davis KE (2009). Coronal alignment in total knee arthroplasty: just how important is it?. J Arthroplasty (Suppl 6).

[CIT0011] Green GV, Berend KR, Berend ME, Glisson RR, Vail TP (2002). The effects of varus tibial alignment on proximal tibial surface strain in total knee arthroplasty: the posteromedial hot spot. J Arthroplasty.

[CIT0012] Hsu HP, Garg A, Walker PS, Spector M, Ewald FC (1989). Effect of knee component alignment on tibial load distribution with clinical correlation. Clin Orthop.

[CIT0013] Huddleston JL, Scott RD, Wimberley DW (2005). Determination of neutral tibial rotational alignment in rotating platform TKA. Clin Orthop.

[CIT0014] Ishii Y, Ohmori G, Bechtold JE, Gustilo RB (1995). Extramedullary versus intramedullary alignment guides in total knee arthroplasty. Clin Orthop.

[CIT0015] Jakob RP, Haertel M, Stussi E (1980). Tibial torsion calculated by computerised tomography and compared to other methods of measurement. J Bone Joint Surg (Br).

[CIT0016] Ko PS, Tio MK, Ban CM, Mak YK, Ip FK, Lam JJ (2001). Radiologic analysis of the tibial intramedullary canal in Chinese varus knees. Implications in total knee arthroplasty. J Arthroplasty.

[CIT0017] Le Damany P (1909). La torsion du tibia, normale pathologique. experimentale. J Anat Physiol.

[CIT0018] Lützner J, Krummenauer F, Günther KP, Kirschner S (2010). Rotational alignment of the tibial component in total knee arthroplasty is better at the medial third of tibial tuberosity than at the medial border. BMC Musculoskelet Disord.

[CIT0019] Maestro A, Harwin SF, Sandoval MG, Vaguero DH, Murcia A (1998). Influence of intramedullary versus extramedullary alignment guides on final total knee arthroplasty component position: a radiographic analysis. J Arthroplasty.

[CIT0020] Mihalko WM, Krackow KA (2006). Differences between extramedullary, intramedullary and computer-aided surgery tibial alignment techniques for total knee arthroplasty. J Knee Surg.

[CIT0021] Mizu-uchi H, Matsuda S, Miura H, Hikagi H, Okazaki K, Iwamoto Y (2006). The effect of ankle rotation on cutting of the tibia in total knee arthroplasty. J Bone Joint Surg (Am).

[CIT0022] Morawa LG, Manley MT, Edidin AA, Reilly DT (1996). Transesophageal echocardiographic monitored events during total knee arthroplasty. Clin Orthop.

[CIT0023] Nagamine R, Miura H, Bravo C, Urabe K, Matsuda S, Miyanishi K, Hirata G, Iwamoto Y (2000). Anatomic variation should be considered in total knee arthroplasty. J Orthop Sci.

[CIT0024] Parker Vail T, Lang JE (2006). Surgical techniques and instrumentation in total knee arthroplasty. In: Insall and Scott surgery of the knee (Ed Scott W N). Churchill Livingstone Elseviere Philadelphia.

[CIT0025] Perillo-Mercone A, Taylor M (2007). Effect of varus /valgus malalignment on bone strains in the proximal tibia after an explicit finite element study. J Biomech Eng.

[CIT0026] Reed MR, Bliss W, Sher JL, Emmerson KP, Jones SM, Partington PF (2002). Extramedullary or intramedullary tibial alignment guides: a randomised, prospective trial of radiological alignment. J Bone Joint Surg (Br).

[CIT0027] Schneider M, Heisel C, Aldinger PR, Breusch SJ (2007). Use of palpable tendons for extramedullary tibial alignment in total knee arthroplasty. J Arthroplasty.

[CIT0028] Takai S, Sakakida K, Yamashita F, Suzu F, Izuta F (1985). Rotational alignment of the lower limb in osteoarthritis of the knee. Int Orthop.

[CIT0029] Teter KE, Bregman D, Colwell C W (1995). Jr. Accuracy of intramedullary versus extramedullary tibial alignment cutting systems in total knee arthroplasty. Clin Orthop.

